# The Beneluxa Initiative domain task force health technology assessment: a comparison of member countries’ past health technology assessments

**DOI:** 10.1017/S0266462323000338

**Published:** 2023-06-15

**Authors:** Rick A. Vreman, Daan van Hoof, Anna Nachtnebel, Joël Daems, Marc van de Casteele, Emer Fogarty, Roisin Adams, Lonneke Timmers

**Affiliations:** 1 National Health Care Institute (Zorginstituut Nederland, ZIN), Diemen, The Netherlands; 2 Division of Pharmacoepidemiology and Clinical Pharmacology, Utrecht Institute for Pharmaceutical Sciences (UIPS), Utrecht University, Utrecht, The Netherlands; 3 Austrian Social Insurance (Dachverband der österreichischen Sozialversicherungen, DVSV), Vienna, Austria; 4 National Institute for Health Insurance and Disability (RIZIV-INAMI), Brussels, Belgium; 5 National Centre for Pharmacoeconomics, Dublin, Ireland; 6 Department of Pharmacology and Therapeutics, School of Medicine, Trinity College Dublin, Dublin, Ireland

**Keywords:** health technology assessment, Beneluxa, Beneluxa Initiative, DTF-HTA, comparative analysis, HTA systems science, international collaboration, added benefit, relative effectiveness assessment, cost-effectiveness assessment

## Abstract

**Objective:**

This study aimed to compare assessments between Beneluxa Initiative member countries’ assessments and identify alignments and divergences.

**Methods:**

A retrospective comparative analysis was performed that investigated (i) number and type of assessed indications (for Austria (AT), Belgium (BE), Ireland (IE), and the Netherlands (NL)); (ii) added benefit conclusions (for BE, IE, and NL); and (iii) the main arguments underlying differences in conclusions (for BE, IE, and NL). Data were retrieved directly from agency representatives and from public HTA reports. European Medicines Agency approved indications were included for drugs assessed between 2016 and 2020, excluding veterinary drugs, generics, and biosimilars.

**Results:**

Only 44 (10 percent) of the 444 included indications were assessed by all four member countries. Between any pair of two countries, the overlap was higher, from 63 (AT–NL) to 188 (BE–IE). Added benefit conclusions matched exactly in 62–74 percent of the indications, depending on the countries compared. In the remaining cases, most often a difference of one added benefit level was observed (e.g., higher vs. equal relative effect). Contradictory outcomes were very rare: only three cases were observed (lower vs. higher effect). When assessing the underlying arguments for seven cases with different outcomes, differences were attributable to slight differences in weighing of evidence and uncertainties rather than disagreement on aspects within the assessment itself.

**Conclusions:**

Despite high variability in European HTA procedures, collaboration on HTA between the Beneluxa Initiative member countries is very feasible and would likely not result in added benefit conclusions that would be very different from added benefit conclusions in national procedures.

## Introduction

Differences between national health technology assessment (HTA) methods, practices, and procedures have led to differences in HTA outcomes (1–3). Simultaneously, HTA practices share the same common elements (e.g., relative effectiveness assessments) and when countries conduct these evaluations separately this leads to duplication of work (4). Consequently, international collaboration can offer substantial advantages. The European network for HTA (EUnetHTA) was founded to promote more effective use of HTA resources, to reduce duplications of HTA, and to increase the overall HTA impact (5–7).

Although EUnetHTA is mostly focused on the clinical domains of HTA, it has been acknowledged that cooperation beyond the clinical domains of HTA may be helpful, including pharmacoeconomic assessment and joint pricing negotiations. Consequently, the Beneluxa Initiative was founded in 2015 to explore possible collaboration on pharmaceutical policy, including joint HTA and price negotiations for pharmaceuticals. Within this initiative, the Netherlands (NL), Belgium (BE), Luxembourg, Austria (AT), and Ireland (IE) share the collaborative aim for “*sustainable access to, and appropriate use of, medicines in the participating countries.*” They strive to “*increase patients’ access to high quality and affordable treatments*” (8). The intention of the Beneluxa Initiative has always been to collaborate on topics of mutual interest, but simultaneously fit the joint Beneluxa Initiative processes within national procedures. This is distinctly different from EUnetHTA as the assessments conducted within the Beneluxa Initiative automatically qualify as national assessments whereas past assessments of EUnetHTA may or may not be used by national organizations (9). The Beneluxa Initiative is organized in four domain task forces (DTFs) each focusing on a specific domain of collaboration: horizon scanning (DTF-HS), information sharing and policy exchange (DTF-INF), pricing and reimbursement (DTF-PR), and the DTF that is the focus of this study: HTA (DTF-HTA).

In light of performing joint HTAs within the Beneluxa Initiative, knowing to what extent past assessments of these countries have been similar or different is of value. Scientific literature provides insights into differences between HTA organizations, leading also to differences in reimbursement recommendations by countries for the same assessed drugs (10;11). Differences may stem from assessment processes that are different, from appraisals that weigh different values and uncertainties against each other, as well as from differences in country-specific characteristics such as the political field and organizational structure (2;12).

In order to further develop the processes for joint HTAs, the objective of this study was to compare past assessments of Beneluxa Initiative member countries. The Beneluxa Initiative DTF-HTA includes all aspects of HTA (i.e., including cost-effectiveness assessments), but the focus of this manuscript is on the differences and similarities in added benefit assessments.

## Methods

### Research questions

The main research question was divided into three sub-questions: (i) Which drugs and indications were assessed by which countries and what were the characteristics of the indications assessed by more than one country (called *scope*). (ii) To what extent do conclusions on added benefit correspond and/or differ between countries (called *conclusions*). (iii) What are the similarities and differences in the main arguments that underpin the conclusions for a subset of assessments that had different conclusions (called *argument*s)?

Appendix A of the Supplementary Material briefly summarizes the scope of assessments for each country. More information on processes and practices of each individual organization can be found on their respective websites. Relevant links are also provided in Appendix A of the Supplementary Material.

### Inclusion

A retrospective analysis based on publicly available information was performed for the three research questions related to *scope, conclusions,* and *arguments*, comparing past national assessments of Beneluxa Initiative member countries. The retrospective analysis included HTA reports for drugs assessed between 2016 and 2020 by Austria (DVSV), Belgium (RIZIV-INAMI), Ireland (NCPE), and the Netherlands (ZIN). Assessments from 2015 were also included when another country assessed that same drug-indication combination between 2016 and 2020. Luxembourg is part of the Beneluxa Initiative but currently has no national HTA process and is therefore not included separately in this study.

Drugs approved by the European Medicines Agency (EMA) that were assessed by the member countries in the set timeframe were included. Veterinary drugs, generics, and biosimilars were excluded. The study focused on drug-indication combinations rather than drugs, because certain drugs can be used to treat multiple indications and subgroups may be defined in the HTA process.

### Data collection

Basic information about characteristics of the drugs were gathered from the EMA website, including therapeutic category, Anatomical Therapeutic Chemical classification (ATC), orphan status, conditional approval, exceptional circumstances, accelerated assessment, ATMP status, and date of marketing authorization. Information on first-in-class status of the drugs were included using data from the FDA.

For the analysis of the *scope*, lists of assessed drug-indication combinations for the study period 2015–2020 were provided by all Beneluxa member countries. These lists included Irish rapid review assessments. The indications were included on the HTA level, that is, when the HTA organization had split the EMA indication into multiple different sub-indications and assessed each of them separately, these were included separately in this study.

Data for the analysis of *conclusions* were collected from the full HTA reports following a standardized data extraction form which was based on previously established procedures (11;13;14). The HTA reports from the Netherlands and Ireland were available via the websites from the HTA agencies. The Belgian reports were provided by a representative from the HTA agency in Belgium. Austria was excluded from the analysis of conclusions because Austrian HTA reports are confidential. Only full HTA assessments that included an added benefit conclusion were included, thus excluding among others rapid review assessments from Ireland. To be able to make comparisons, only drug-indication combinations that were assessed by two or more countries were included for the analysis of conclusions. When a specific conclusion was not explicitly stated, it was interpreted from the written text in the reports.

Lastly, data for the analysis of *arguments* were extracted from the (summary of the) full assessment reports of each organization (excluding Austria) following a standardized data extraction form. The data on conclusions and arguments were extracted by one author and a 10 percent subset was validated by organizations’ representatives.

### Analyses

The analysis of the scope was a descriptive analysis, listing the number of assessed drug-indication combinations per country and across countries. Tables were constructed that showed the overlap in drug-indication combinations with seven characteristics, being: therapeutic category (based on ATC code), orphan status, conditional approval, approval under exceptional circumstances, accelerated assessment, ATMP status, and first-in-class status.

Added benefit conclusions were categorized into higher, equal, and less therapeutic value which facilitates comparison and is in line with previous studies on this topic (11;15). Similarities between countries in added benefit conclusions were examined by setting one country as the reference through which the conclusions from the other country were compared.

For the analysis of arguments, a set of cases with different conclusions in terms of added benefit and assessed by three countries (BE–IE–NL) were selected. The PICOTE method was used to study differences and similarities (16;17). The PICOTE method looks into the patient population (P), the intervention (I), comparator (C), outcomes (O), timing (T), and included evidence and its weighing (E). This method allows to systematically investigate on which issues or uncertainties within these domains the countries agree or differ in opinion. Case studies were analyzed by first systematically extracting each of the elements of PICOTE from the assessment report of each country. Subsequently, the elements were individually compared between countries. Differences for each of the elements were then described narratively.

## Results

### Analysis of scope

After applying the exclusion criteria based on the list of approvals of the EMA and splitting the assessed drugs into assessed drug-indication combinations, the data set contained 361 different drugs with 444 indications. Because indications are defined differently by the countries in multiple cases and because not all drugs are assessed by each country, none of the countries comes close to assessing all 444 unique drug-indication combinations. Austria assessed 168 drug-indication combinations, Belgium 335, Ireland 260, and the Netherlands 145 (inclusion flowchart in Appendix B of the Supplementary Material). The overlap in assessed indications between countries in the time period 2016–2020 is demonstrated in Table 1. In total, there were 278 unique drug-indication combinations that were assessed by at least two countries, 142 drug-indication combinations that were assessed by at least three countries, and a total of 44 (10 percent) drug-indication combinations that were assessed by all four countries.

**Table 1.** Overlap in assessed drug-indication combinations (out of *n* = 444) between countries

	The Netherlands	Belgium	Ireland	Austria
The Netherlands	145			
Belgium	113	335		
Ireland	94	188	260	
Austria	63	128	107	168
Belgium–Ireland–Austria	89			
The Netherlands–Belgium–Ireland	79			
The Netherlands–Ireland–Austria	49			
The Netherlands–Belgium–Austria	56			
Four countries	44			

The extent to which specific drug-indication combinations (following six characteristics) were assessed by each country is also shown in Appendix B of the Supplementary Material. The scope of assessments for Austria is limited to outpatient drugs, explaining the lack of overlap in assessed ATMPs. However, the overlap is also small for the other characteristics. Except first-in-class, for every characteristic the overlap of drugs-indication combinations assessed by all countries is less than 10 percent (see Appendix C of the Supplementary Material). The most common cause for this small overlap is the lack of overlap between the Netherlands and Austria.

### Analysis of conclusions

After the exclusion of drug-indication combinations for which a full HTA (excluding rapid reviews) report including a conclusion on added benefit for matching indications was not available for at least two (out of three) countries, 117 drug-indications remained. Belgium assessed 110 indications, Ireland 71, and the Netherlands 80. Overlap existed in seventy-eight indications between Belgium and the Netherlands, sixty-four between Belgium and Ireland, and thirty-nine between Ireland and the Netherlands. Thirty-two indications had conclusions on added benefit (with a full HTA report available) by all three organizations. Thus, from the seventy-nine indications assessed by Belgium, Ireland, and the Netherlands (see Table 1), about 60 percent (*n* = 47) had no full HTA report including an added benefit conclusion in at least one country, mostly due to the exclusion of the rapid review assessments of Ireland.

**Figure 1.**
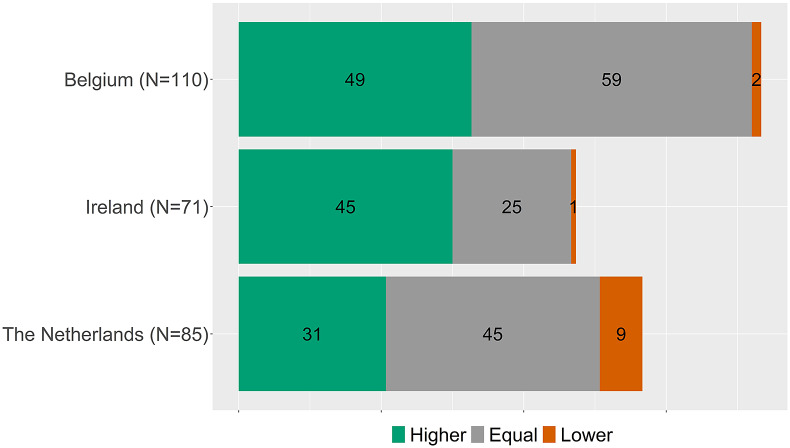
Overview of added benefit conclusions per country.

**Figure 2.**
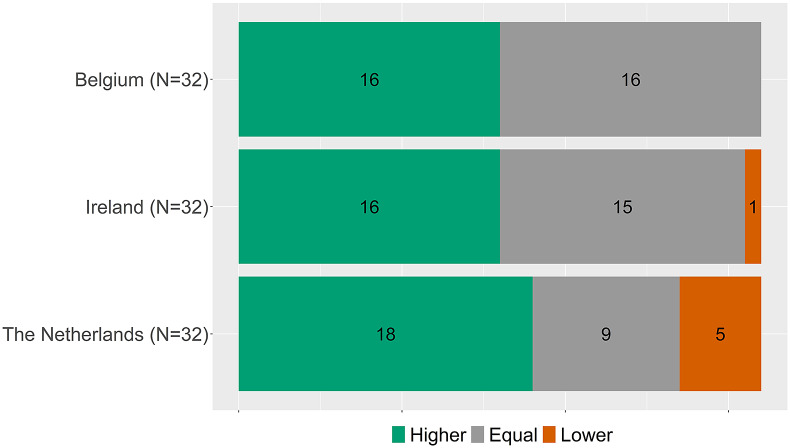
Overview of added benefit conclusions per country for the thirty-two drug-indication combinations assessed by all three countries.

Figure 1 shows the added benefit conclusions per country. To facilitate direct comparison, Figure 2 shows the added benefit conclusions from the countries of the subset of the thirty-two drug-indication combinations assessed by all three countries.

### Conclusions for matched indications

**Figure 3.**
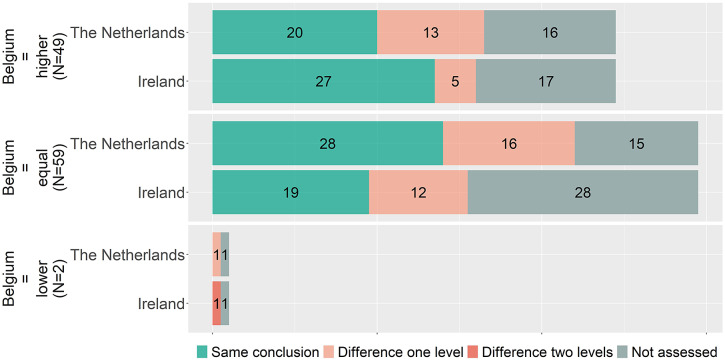
Added benefit conclusions from Belgium (*N* = 110) compared to the Netherlands and Ireland. Not assessed indicates that for those conclusions of Belgium, no conclusion in a full health technology assessment (HTA) report for a matching indication was available from Ireland or the Netherlands, respectively.

**Figure 4.**
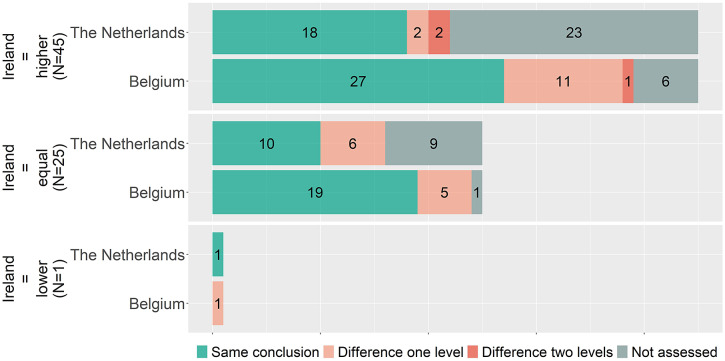
Added benefit conclusions from Ireland (*N* = 71) compared to Belgium and the Netherlands. Not assessed indicates that for those conclusions of Ireland, no conclusion in a full health technology assessment (HTA) report for a matching indication was available from Belgium or the Netherlands, respectively.

**Figure 5.**
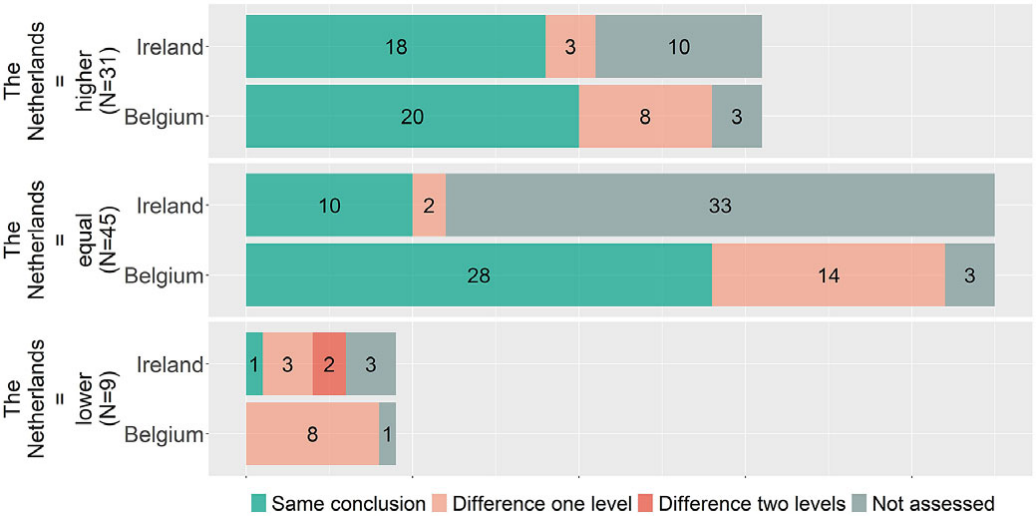
Added benefit conclusions from the Netherlands (*N* = 85) compared to Belgium and Ireland. Not assessed indicates that for those conclusions of the Netherlands, no conclusion in a full health technology assessment (HTA) report for a matching indication was available from Ireland or Belgium, respectively.

Figure 2 indicates that overall proportions of higher, equal, and lower benefit ratings are similar, but it does not show whether for individual indications the conclusions matched. To explore the coherence in individual indications, the conclusions are matched per indication between countries. Accordingly, Figures 3–5 show, for matched indications, the conclusions of the two other countries from the perspective of the conclusion of the first country. For example, when one country concludes in ten cases a higher added benefit, it is shown for the other two countries on how many of those ten they also came to a higher benefit conclusion. The results are classified as matching conclusions (in green), conclusions differing by one level (e.g., higher vs. equal added benefit, in yellow), and conclusions differing by two levels (higher vs. lower, in orange). Overall, conclusions matched exactly between Belgium and the Netherlands in forty-eight out of seventy-eight (62 percent) matched indications, in forty-six out of sixty-four (72 percent) indications between Belgium and Ireland, and in twenty-nine out of thirty-nine (74 percent) indications between Ireland and the Netherlands. Contradictory conclusions were very rare: none between Belgium and the Netherlands, only one between Belgium and Ireland, and two between Ireland and the Netherlands.

### Analysis of arguments

Out of the thirty-two drug-indication combinations that were assessed by three countries (BE, IE, and NL, see Figure 2), seven (22 percent) drug-indications had different added benefit conclusions and were thus included in the qualitative in-depth analysis (olaparib, selexipag, migalastat, teduglutide, lesinurad, rivaroxaban, and tisagenlecleucel). The detailed case study descriptions can be found in Appendix D of the Supplementary Material. Following the PICOTE method, no differences in highlighted issues or uncertainties regarding the patient population (P), the intervention (I), or the relevant outcome measures (O) were noted. Only in one case the comparator (C) differed between jurisdictions (selexipag). The timing (T) of some assessments differed between countries (selexipag and teduglutide). The main evidence was always the same but in some cases additional supportive studies were included by one organization and not the others (migalastat, selexipag, teduglutide, and lesinurad). The differences in added benefit conclusions could in all but one case (selexipag, because of the different comparator) be interpreted as the result of country-specific weighing of the clinical data and associated uncertainties of the included evidence and contextual considerations.

## Discussion

When investigating all assessed drug-indication combinations in 2016–2020, it was found that there was variability mainly due to the different scopes of the organizations in the drugs that they assess (see Appendix A of the Supplementary Material). This resulted in a small level of overlap of indications assessed by all countries: only forty-four (10 percent). The overlap between two or three countries was substantially higher. The overlap in indications assessed that were associated with specific characteristics was below 10 percent for all included characteristics (therapeutic category, orphan status, conditional approval, exceptional circumstances, accelerated assessment, and ATMP), only for first-in-class drugs the overlap was above 10 percent. Thus, it can be concluded that we did not find certain drugs or groups of drugs that were assessed by all countries. Explanations for this finding are the different inclusion- and exclusion criteria that the countries use for performing HTAs and the fact that the manufacturer is in control of where to submit at what time and for which indications. The implication of this finding is that it might be helpful to establish joint assessment selection criteria that take into account the (sometimes legal) scope of each country, and to investigate in more detail why assessed indications differ and whether that could be resolved. Within the Beneluxa Initiative joint assessment process, the indication is jointly defined so the differences in assessed indications should not lead to difficulties within Beneluxa joint HTAs.

The analysis of the agreement on the conclusions shows that overall, Belgium, Ireland, and the Netherlands mostly agree on aspects included in added benefit assessments (i.e., PICOTE). Conclusions are rarely contradictory. However, in a minority of cases conclusions do indeed differ. The in-depth case analysis showed that the variation in added benefit conclusions can be mainly attributed to the difference in weighing and interpretation of the clinical data and associated uncertainties. The analysis showed that this was in most cases due to unclear, contradicting or low quality of the evidence included. This suggests that the larger evidence gaps are, the more negative impact it may provoke on HTA alignment between countries. This might have been expected, as when the underlying evidence base is scarce and uncertainties high, deriving a conclusion on the added benefit poses challenges to member countries and therefore these situations are more likely to lead to differences. Nonetheless, especially these circumstances also offer the highest benefit for collaboration, as closer interaction for example through more information sharing between committees that form added benefit conclusions can mitigate some of these differences.

The results of this study are somewhat contrasting to other studies that compare outcomes of HTAs between countries (1;2;10;11;14;18–20). Often, studies comparing HTA outcomes find that they are very variable between countries. Several explanations might be put forward for the higher agreement found in this study. First, countries within the Beneluxa Initiative are relatively similar in how advanced their HTA systems are. Second, we compared added benefit assessment outcomes only for indications that matched between countries, whereas some studies compare assessments directly without excluding indications that are not equal or at least similar. Our approach limits the sample size but gives a more appropriate insight into the level of agreement. Similarly, some studies report the overall HTA recommendation rather than solely the REA, which means their results can also include economic aspects and therefore include additional discrepancies between countries beyond the REA.

The Beneluxa Initiative is one example of a regional collaboration on HTA. In Europe, the most important international collaboration is EUnetHTA. The objectives of the Beneluxa Initiative are explicitly broader than the scope of EUnetHTA, and include horizon scanning, joint assessment of economic domains, and joint negotiations, however, these assessments are not part of this first paper from the Beneluxa Initiative. In line with the scope of EUnetHTA, the EU HTA regulation to be implemented in 2025 is for joint assessments limited to the clinical domains of the EUnetHTA Core Model, although the regulation leaves room for EUnetHTA to facilitate voluntary cooperation beyond these domains. The Beneluxa Initiative is involved in, and welcomes, dialogue with other regional initiatives on the domains that are part of the Beneluxa Initiative. For example, the International Horizon Scan Initiative, which is a collaboration of nine countries, has its roots in the Beneluxa collaboration on HS. With the adoption of the EU HTA Regulation, a new phase in European collaboration is starting, and regional collaboration, including the Beneluxa Initiative, can further develop their way of working to avoid duplication (21). The authors of this paper hope that publications about the Beneluxa Initiative such as this study will inspire the initiation or development of other (regional) HTA collaborations.

### Limitations

Some limitations may have had an impact on the results. We set a specific timeframe for inclusion, but manufacturer submissions are not synchronized between countries, meaning that we might have excluded some indications that were not yet assessed in one or more countries. Additionally, the HTA reports did not always provide a clear categorization or conclusion on the added benefit (i.e., it may be included in the overall conclusion but not explicitly reported separately). If this was the case, the combinations were categorized following author interpretation. Ten percent of these interpretations were validated by agency representatives and found to be 100 percent correct, suggesting the impact of these interpretations may have been negligible. The (sometimes legal) scope of HTA within each of the countries had an impact on the *scope* analysis. The organizations’ scopes are summarized in Appendix A of the Supplementary Material. HTA reports from Austria had to be excluded for the second and third part (conclusions and arguments) of the research because they are not publicly available. Consequently, the results from the analysis of the conclusions and the arguments analysis may not be applicable to the process and outcomes from Austria, and differences between all four of the included member countries of the Beneluxa Initiative may actually be larger than reported for the three member countries in this study. This study does not include any information on cost-effectiveness outcomes or other aspects of HTA. Although this is within the scope of the Beneluxa Initiative, outcomes of cost-effectiveness assessments are much harder to quantitatively compare due to national differences in costs, thresholds, and other aspects within cost-effectiveness analyses.

## Conclusions

Between Beneluxa Initiative member countries, the drugs that are assessed in national procedures and the definition of the assessed indications show relatively large differences. However, conclusions on added benefit were mostly in agreement. When conclusions differed, they could generally be explained by slight differences in weighing of evidence and uncertainties. Overall, it can be concluded that collaboration on HTA between the Beneluxa Initiative member countries would likely not result in added benefit conclusions that would be different from added benefit conclusions in national procedures.

## Supplementary material

For supplementary material accompanying this paper visit https://doi.org/10.1017/S0266462323000338. click here to view supplementary material
