# Supplementary material for: The Beneluxa Initiative domain task force health technology assessment: a comparison of member countries’ past health technology assessments
Source: Int J Technol Assess Health Care. 2023 Jun 15;39(1):e44. doi: 10.1017/S0266462323000338 (PMC11570237; doi:10.1017/S0266462323000338)
Supplement: Supplementary file 1 [file S0266462323000338sup001.docx]

**Appendices**

**Appendix A. Brief summaries of country scopes of assessment.**

*Scope*

The national scopes of drugs that are selected for assessment differ widely.

Austria: assesses outpatient drugs, no inpatient drugs.

Belgium: assess both inpatient and outpatient drugs.

Ireland: starts with a rapid review for all drugs (both inpatient and outpatient drugs). If the drug does not meet the criteria, a full HTA assessment is done.

The Netherlands: assess all outpatient drugs and a selection of inpatient drugs based on high budget impact and costs per patient criteria.

Websites for more information on processes:

The Beneluxa Initiative DTF-HTA: <https://beneluxa.org/HTA/>

Austria: <https://www.sozialversicherung.at/cdscontent/?contentid=10007.859105&portal=svportal>

Belgium:<https://www.inami.fgov.be/nl/riziv/organen/Paginas/commissie-tegemoetkoming-geneesmiddelen.aspx> (Dutch)

<https://www.inami.fgov.be/fr/inami/organes/Pages/commission-remboursement-m%C3%A9dicaments.aspx> (French)

Ireland: <https://www.ncpe.ie/submission-process/>

The Netherlands: <https://english.zorginstituutnederland.nl/about-us/tasks-of-the-national-health-care-institute>

**Appendix B. Inclusion flowchart and characteristics.**

Supplementary Figure 1: Inclusion flowchart.

**
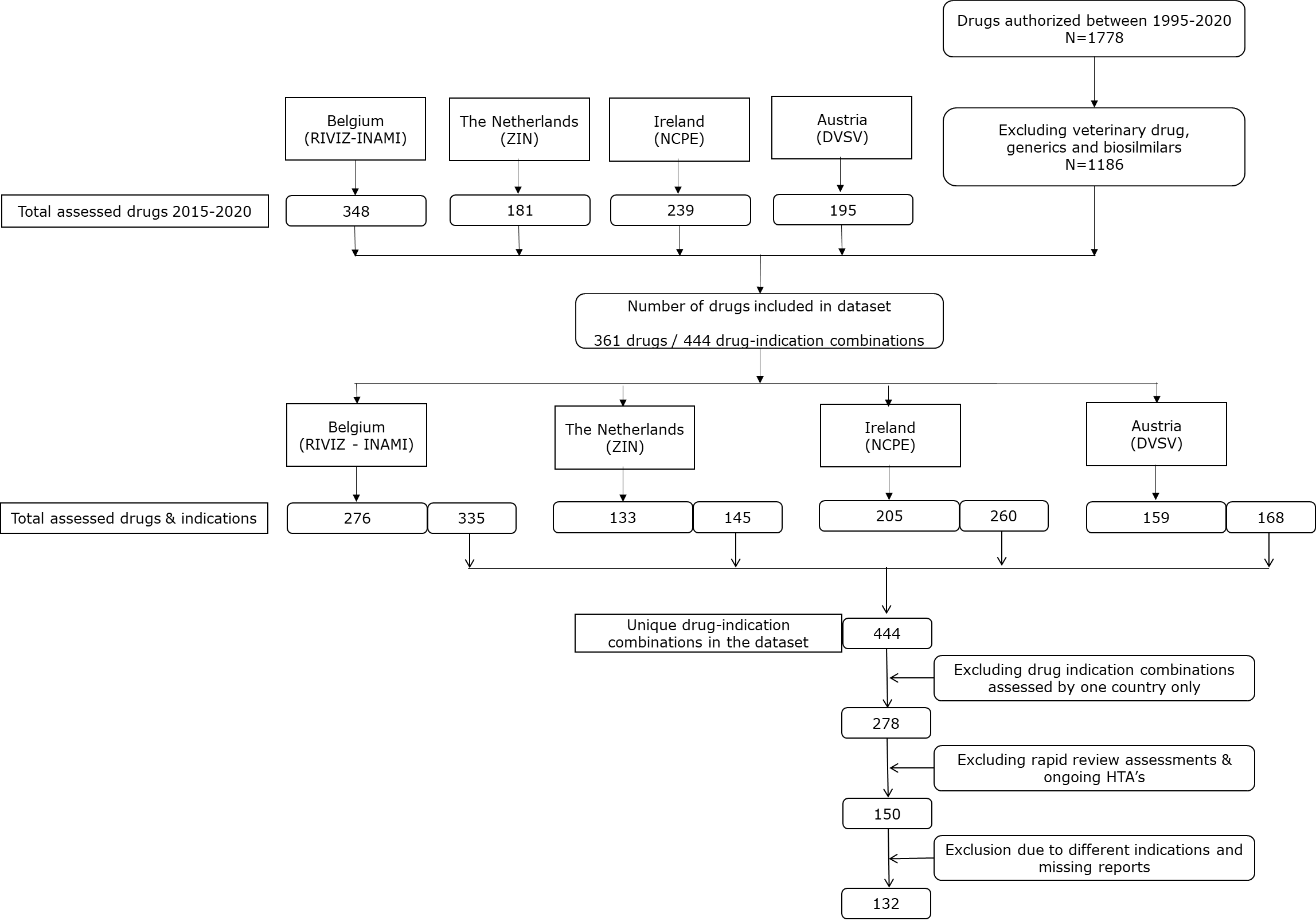
**

*Supplementary Table 1: characteristics of the drug-indication combinations per country.*

|  | AT | BE | IE | NL | Total throughout all countries |
| --- | --- | --- | --- | --- | --- |
| Orphan | 25 | 75 | 65 | 39 | 98 |
| Conditional marketing authorization | 5 | 12 | 10 | 4 | 15 |
| Authorization under exceptional circumstances | 3 | 11 | 6 | 8 | 15 |
| Accelerated assessment | 13 | 34 | 28 | 17 | 44 |
| First-in-class | 17 | 31 | 27 | 17 | 38 |
| Advanced therapy medicinal product | 0 | 7 | 7 | 4 | 8 |

**Appendix C. Characteristics of included indications.**

*Supplementary Table 2: overlap in assessed drug-indication combinations between the countries based on ATC group.*

|  | AT-BE-IE | AT-BE-NL | AT-IE-NL | BE-IE-NL | All four | Total drug-indication combinations |
| --- | --- | --- | --- | --- | --- | --- |
| A: Alimentary tract and metabolism | 10 | 11 | 9 | 14 | 8 | 53 |
| B: Blood and blood forming organs | 5 | 4 | 3 | 5 | 3 | 34 |
| C: Cardiovascular system | 5 | 4 | 5 | 5 | 4 | 14 |
| D: Dermatologicals | 1 | 0 | 0 | 0 | 0 | 6 |
| G: Genito urinary system and sex hormones | 0 | 0 | 0 | 0 | 0 | 4 |
| H: Systemic hormonal preparations, excluding sex hormones and insulins | 0 | 0 | 1 | 0 | 0 | 6 |
| J: Anti-infective for systemic use | 12 | 14 | 13 | 15 | 12 | 51 |
| L: Antineoplastic and immunomodulating agents | 45 | 13 | 11 | 23 | 11 | 201 |
| M: Musculo-skeletal system | 2 | 1 | 1 | 3 | 1 | 12 |
| N: Nervous system | 5 | 5 | 3 | 8 | 3 | 31 |
| R: Respiratory system | 4 | 3 | 2 | 3 | 1 | 18 |
| S: Sensory organs | 0 | 0 | 0 | 1 | 0 | 6 |
| V: Various | 1 | 1 | 1 | 1 | 1 | 6 |

*Supplementary Table 3: overlap in assessed drug-indication combinations between the countries based on characteristics of the drug-indication combination*

|  | AT-BE-IE | AT-BE-NL | AT-IE-NL | BE-IE-NL | All four | Total drug-indication combinations |
| --- | --- | --- | --- | --- | --- | --- |
| Orphan | 11 | 11 | 8 | 23 | 6 | 98 |
| Conditional marketing authorization | 4 | 1 | 2 | 2 | 1 | 15 |
| Authorization under exceptional circumstances | 0 | 3 | 0 | 2 | 0 | 15 |
| Accelerated assessment | 8 | 5 | 3 | 9 | 3 | 44 |
| First-in-class | 12 | 10 | 8 | 12 | 8 | 38 |
| Advanced therapy medicinal product | 0 | 0 | 0 | 4 | 0 | 8 |

**Appendix D. Detailed case study descriptions.**

*Each of the case studies was analysed following the PICOTE (population, intervention, comparator, outcomes, timing and evidence) framework, but emphasis in each case study description is placed mostly on the domain where differences were present (therefore not in each case study each element is discussed). The domains are listed within parenthesis.*

*Olaparib (Lynparza)*

Olaparib (*intervention*) is indicated for the maintenance treatment of adult patients with advanced (FIGO stages III and IV) BRCA1/2-mutated (germline and/or somatic) high-grade epithelial ovarian, fallopian tube or primary peritoneal cancer who are in response (complete or partial) following completion of first-line platinum-based chemotherapy (*population*).

All countries included the SOLO1-trial (*evidence*) by Moore et al. (2018) which used a placebo (watch and wait) as a comparator (*comparator*). Both Belgium and the Netherlands mention in the analysis of the trial that olaparib has a clinically relevant and statistically significant increase of progressive free survival (PFS). Nevertheless, both countries mentioned the immaturity of the data on outcome measures such as health related quality of life and overall survival which made it harder to draw conclusions (*outcomes*). Overall, the Netherlands and Belgium qualified olaparib as a drug that has a therapeutic added value. Ireland reported similar uncertainties but reported that an added benefit could not be concluded.

*Selexipag (Uptravi)*

Selexipag (*intervention*) is indicated for the long-term treatment of pulmonary arterial hypertension (PAH) in adult patients with WHO functional class (FC) IIIII, or as combination therapy in patients inadequately controlled with an endothelin receptor antagonist (ERA) and / or a phosphodiesterease type. 5 (PDE-5) inhibitor, or as monotherapy in patients not eligible for these treatments (*population*).

Selexipag was assessed by NL (07-2016), Ireland (05-2018), and Belgium (12-2020) (*timing)*. The added benefit conclusions differed between Belgium and the Netherlands and Ireland. All countries included the GRIPHON study by Sitbon et al. (2015) which was a double-blind placebo-controlled phase 3 study (*evidence*). All countries mention the significant effect on the primary end-point of the study and the non-significant effect on mortality (*outcomes*). As a result, Belgium came to the overall conclusion of added therapeutic benefit based on the added benefit on quality of life and morbidity. The Netherlands and Ireland came to another conclusions which seems to be the result of the chosen comparator. The Netherlands chose bosentan as a relevant comparator (*comparator*). In their analysis they do agree on the fact that selexipag is more effective than the placebo. However, they do not find an added therapeutic benefit compared to bosentan, which was already reimbursed at the time. Therefore, the Netherlands did not grant added therapeutic benefit to selexipag. Ireland used inhaled iloprost as a comparator (*comparator*). In their analysis they mentioned that there is no evidence to demonstrate improved persistence and adherence with selexipag compared with inhaled iloprost. Thereby, they raised questions on the statistically and clinically relevant effects found because they mentioned that interpretation of results of the composite endpoint is challenging when individual components are not associated with equal importance.

*Migalastat (Galafold)*

Migalastat (*intervention*) is indicated for the treatment of Fabry disease in patients with an amenable mutation (*population*).

All countries based their conclusions on two phase III studies: the first randomized controlled trial, called ATTRACT, compared open label migalastat versus continued enzyme supplementation in pre-treated patients. The second randomized controlled trial was a double blind trail called FACETS that looked into treatment-naïve patients and compared migalastat versus placebo (*evidence*).

All countries described that the clinical data from the included trial does not result in clear added benefit compared to enzyme replacement therapy which is used as comparator in all countries (*comparator*). Belgium and Ireland conclude that migalastat has a similar efficacy when compared to enzyme replacement therapy. On the other hand, the Netherlands conclude that migalastat has a lower therapeutic value when compared to enzyme replacement therapy based on insufficient data.

*Teduglutide (Revestive)*

Teduglutide (*intervention*) is indicated for the treatment of patients aged 1 year and older with short bowel syndrome (SBS), provided the patient is stable after surgery and after a period of intestinal adjustment (*population*).

All countries included the STEPS studies (STEPS1 and STEPS2) and the TED-C13-003 trial for the assessment of the therapeutic value of teduglutide (*evidence*). Besides that, all countries used placebo as the comparator (*comparator*). The trials show a reduction in parenteral support (more than 20% volume reduction) in the patient group that used teduglutide compared to the placebo group (*outcomes*). None of the included studies showed a statistically significant difference between placebo and teduglutide on the different quality of life scores (SF-36, EQ-5D and IBDQ) (*outcomes*).

As a result, the Netherlands concluded on a lower therapeutic value for teduglutide when compared to a placebo. They mention that – even though the reduction in parenteral support was found – the patient remains partly dependent on parenteral nutrition (*outcomes*). Moreover, it is not clear to what extent the measured effects can be attributed to natural adaptation. That in combination with the fact that no difference in quality of life could be demonstrated between the group treated with teduglutide and the control group, resulted in the negative conclusion. Belgium does not precisely report the considerations regarding the negative therapeutic value in the report. However, in the second assessment it is mentioned that the first assessment had a negative conclusion. Ireland on the other hand mainly focused on the decrease of parenteral support which is in their opinion a positive effect.

*Lesinurad (Zurampic)*

Lesinurad (*intervention*) is indicated for the treatment of hyperuricaemia in adult patients with gout who have not achieved target serum uric acid levels with an adequate dose of a xanthine oxidase inhibitor (*population*).

For the assessment of lesinurad, all countries included the CLEAR-1 and CLEAR-2 trials in combination with the CRYSTAL study (*evidence*). As an addition, the Netherlands included a study by Reinders (2009) which looked into the effects of allopurinol and benzbromaron. All countries agree on the fact that the data do not suggest a therapeutic added benefit for lesinurad over current therapy with allopurinol or febuxostat as monotherapy (*comparator*).

Even though the countries agree, the added benefit conclusions differ. The Netherlands concluded a loss of therapeutic value due to insufficient data on the effectiveness. Ireland phrases the data from the trials differently: the clinical trials did not show any significant difference in quality of life with lesinurad in combination with a xanthine oxidase inhibitor as compared with xanthine oxidase monotherapy (*outcomes*). Therefore, there is no additional benefit of lesinurad plus a xanthine oxidase inhibitor over current second line therapy (febuxostat monotherapy). They did not state a lower therapeutic value. Last, Belgium mentions that they cannot grant a therapeutic added benefit to lesinurad but did not conclude on a lower therapeutic value. In their analysis they briefly addressed a result from an informal indirect comparison of the pooled results for the two CLEAR studies with the second-line population in a long-term study of febuxostat which showed a very similar response rate in all cases

*Rivaroxaban (Xarelto)*

Rivaroxaban (*intervention*) is indicated for the prevention of atherothrombotic events in adult patients with coronary artery disease (CAD) or symptomatic peripheral artery disease (PAD) at high risk of ischaemic events (*population*).

The main study for the assessment of rivaroxaban is the COMPASS study which was a double-blind trial to assess the effectiveness and safety of rivaroxaban. All countries included the COMPASS study and the Netherlands included two extra studies that were based on the COMPASS study (*evidence*). The comparator in the study was aspirin (acetylsalicylic acid) which was also the relevant comparator considered by all countries (*comparator*). Findings from the study were valued differently by the countries in terms of added benefit. The COMPASS study shows a positive effect on cardiovascular death, stroke or myocardial infarction when rivaroxaban is combined with acetylsalicylic acid. However, the use of rivaroxaban also resulted in some serious negative side-effects such as more major bleeding events compared to the treatment group that used acetylsalicylic acid alone (*outcomes*). For the Netherlands, the benefits outweigh the possible negative side-effects. Therefore, rivaroxaban has a therapeutic added value over acetylsalicylic acid due to the positive effect on cardiovascular death, myocardial infarction and stroke and on all cause death, as found in the COMPASS study. Belgium and Ireland are more reluctant. Belgium states that the positive effects must be nuanced because it is limited in absolute numbers. Because of the side-effects and unknown therapeutic value in the long-term (over 3 years), Belgium states that the added therapeutic value cannot be proven. Ireland has similar considerations and states that – besides the positive effects on cardiovascular outcomes – the major bleeding events need to be taken into account seriously.

*Tisagenlecleucel (Kymriah)*

Tisagenlecleucel (*intervention*) in this case study was indicated for diffuse large B-cell lymphoma (DLBCL) and follicular lymphoma (FL) in adults whose cancer has come back or did not respond after two or more previous treatments (*population*).

The effectiveness and safety of tisagenlecleucel has been investigated in a single-arm study (C2201). The comparison with chemotherapy is based on two matching adjusted indirect comparisons (MAIC) with historical controls (C2201 vs. SCHOLAR-1 and C2201 vs. CORAL extension) (*comparator*). All countries included these studies to examine the therapeutic value of tisagenlecleucel for DLBCL (*evidence*). All countries come to the same conclusion that the efficacy data is limited, and the quality of the evidence is very low which makes it impossible to conclude if tisagenlecleucel is superior over other treatments such as axicabtagene ciloleucel, rituximab, gemcitabine, dexamethasone, cisplatin, ifosfamide and oxaliplatin (*comparator*). The Netherlands labels tisagenlecleucel with less therapeutic value based on these findings. Ireland does not mention less therapeutic value but highlights the very limited evidence base. Belgium also does not grant superiority to tisagenlecleucel but does not mention less therapeutic value.
